# Evolution of the Surface Wettability of PET Polymer upon Treatment with an Atmospheric-Pressure Plasma Jet

**DOI:** 10.3390/polym12010087

**Published:** 2020-01-03

**Authors:** Alenka Vesel, Rok Zaplotnik, Gregor Primc, Miran Mozetič

**Affiliations:** Department of Surface Engineering, Jozef Stefan Institute, Jamova cesta 39, 1000 Ljubljana, Slovenia; rok.zaplotnik@ijs.si (R.Z.); gregor.primc@ijs.si (G.P.); miran.mozetic@ijs.si (M.M.)

**Keywords:** atmospheric-pressure plasma jet, polymer, surface patterns, wettability mapping, XPS mapping

## Abstract

A useful technique for pre-treatment of polymers for improved biocompatibility is surface activation. A method for achieving optimal wettability at a minimal thermal load and unwanted modifications of the polymer properties is elaborated in this paper. Samples of polyethylene terephthalate polymer were exposed to an atmospheric-pressure plasma jet created by a high-impedance low-frequency discharge in wet argon. Different treatment times and distances from the end of the glowing discharge enabled detailed investigation of the evolution of surface activation. A rather fast saturation of the surface wettability over the area of the order of cm^2^ was observed upon direct treatment with the glowing discharge. At a distance of few mm from the glowing discharge, the activation was already two orders of magnitude lower. Further increase of the distance resulted in negligible surface effects. In the cases of a rapid activation, very sharp interphase between the activated and unaffected surface was observed and explained by peculiarities of high-impedance discharges sustained in argon with the presence of impurities of water vapor. Results obtained by X-ray photoelectron spectroscopy confirmed that the activation was a consequence of functionalization with oxygen functional groups.

## 1. Introduction

Polymers are nowadays widely used in industry, as well as in medicine because of their excellent chemical inertness and mechanical properties. The biocompatibility of most polymers with appropriate mechanical properties is, however, below requirements; therefore, they should be modified prior to incubation with a biological matter. The biocompatibility of materials depends predominantly on surface properties. The best surface properties are obtained by grafting biocompatible molecules, preferably by forming covalent bonds between a monolayer of grafted molecules and the substrate [1,2,3,4,5,6]. Usually, such bonding cannot occur because of the inertness of most polymers of appropriate chemical and mechanical properties. The surface properties of polymers should be, therefore, altered to enable at least reasonable if not excellent biocompatibility. A common method for tailoring surface properties of polymers is a brief exposure to non-equilibrium gaseous plasma. A review of plasma techniques for achieving desired properties has been published recently [7].

Any attempt of grafting biocompatible coatings on polymer substrates require alternation of the surface wettability. The surface composition needs to be modified to achieve desired hydrophilicity, and thus, adhesion properties. Many different plasma configurations can be used to achieve better wettability ranging from low-pressure to atmospheric-pressure plasmas. Nowadays, atmospheric-pressure plasma jets (APPJ) are particularly popular, and their application in polymer surface treatment is still increasing [8,9,10,11]. Such plasmas enable localized treatment of what is beneficial in various biomedical applications [12,13,14,15]. Details about the surface finish, in particular, on gradients of surface properties, however, are rarely reported in the scientific literature. Such details are of crucial importance to understand the surface chemistry and prepare the surface conditions which are regarded as optimal for a particular application. Below are selected papers that have addressed the surface finish, in particular, two-dimensional mapping and/or determination of gradients likely to occur on polymer surfaces upon treatment with atmospheric plasma jets. 

Birer [16] performed a very interesting investigation regarding the reactivity zones formed on polyethylene (PE) surface after treating with an APPJ using helium and argon gas or its mixture with oxygen or nitrogen. The sample was positioned 1 cm away from the nozzle of the discharge tube. Reactivity zones were investigated by two-dimensional X-ray photoelectron spectroscopy (XPS) mapping. He found that oxidation started at the center hit by the plasma jet and then expanded outwards forming a ring-shape pattern. Formation of ring patterns of –NO, –COO, –CO and –NO_3_ groups with diameters increasing with treatment time was detected. Surface modified by APPJ treatment expanded several millimeters from the center. Thus, a diameter of the zone modified by APPJ was found to be up to about 2 cm from the plasma jet axis. Kostov et al. [17] also investigated the wettability as well as surface morphology by atomic force microscopy (AFM) of polymers polyethylene (PE), polypropylene (PP) and polyethylene terephthalate (PET) treated by APPJ operating in argon. Samples were placed at various distances between 2 and 3.5 cm from the nozzle of the APPJ. He provided radial water contact angle profiles and found the similar size of the modified area as Bierer [1] found by XPS mapping-approximately 2 cm of diameter.

Jofre-Reche et al. [18] investigated the effect of the nozzle distance to the polydimethylsiloxane (PDMS) sample surface (in the range up to 10 mm) and found an optimum surface wettability at the distance of 6.6 mm. This correlated well with the lower gas temperature at this distance and higher optical emission intensity (OES) of O (777 nm) line in the plasma jet. Moreover, Deynse et al. [19] investigated the effect of the nozzle distance on the surface wettability of PE treated with Ar plasma jet. For distances up to 15 mm, 70% of increase of surface wettability was found, whereas, at longer distances a sharp knee appeared on the curve of the surface wettability versus the distance. Such variation of the surface wettability corresponded well with the changes in the chemical composition regarding XPS O/C ratio and concentration of various oxygen functional groups. Wagenaars et al. [20] investigated the wettability of PP treated with APPJ in helium with various oxygen admixtures. They found a minimum in the water contact angle (WCA) when 0.5% of oxygen was added to helium. The WCA also depended on treatment time and a distance between the APPJ nozzle and PP sample. A significant decrease of WCA was observed in the first 10 s of treatment for distances up to 20 mm, whereas, later WCA slowly stabilized. For very short distances 3 and 5 mm the WCA stabilized in 40 s. The minimum achievable WCA decreased with increasing distance. Because the O-atom density is lower at longer distances, applying longer treatment time would give the same total flux as in the case of a low distance (high O-atom density) and low treatment time. Therefore, the same final effect should be expected. However, this was not observed; therefore, the authors concluded that O-atom density is important and not the total flux. 

Dowling et al. [21] investigated the influence of DC pulse plasma cycle time (PCT) on the activation of polypropylene (PP), polystyrene (PS) and polycarbonate (PC) polymers. They found that optimized PCT was specific for a given polymer and related to the polymer thermal properties. Lommatzsch [22] was investigating differences in the surface reactions when treating polymer PE with APPJ created in air or nitrogen. 

Some authors have also investigated the effect of water vapor on surface modification of polymers [23,24,25]. Oehrlein et al. [24] have investigated the effect of water vapor in Ar/H_2_O plasma on the etching rates of polymers. He found that OH radicals formed in plasma play a dominant role in the etching process. The etching rates dropped exponentially when a distance between polymer and APPJ was increased what was consistent with a density of OH radicals that also exponentially decreased with a distance. The exponential decay constant for Ar/H_2_O plasma was 3.40 and 6.07 mm for the case of air or nitrogen environment, respectively. Contrary, regarding an oxygen content on the etched surface, a maximum was observed at intermedium distance of a polymer from the APPJ. Moreover, Sarani et al. [25] investigated the effect of water vapor in Ar plasma. They found a higher oxygen content on a polymer surface as measured by XPS for the case when the polymer was treated with Ar/H_2_O plasma jet, what was explained with a higher density of radicals in the jet afterglow. Nevertheless, a difference in XPS oxygen concentration for the case of Ar/H_2_O plasma in comparison to pure Ar plasma is small. 

Foest et al. [26] investigated vacuum ultraviolet (VUV) emission (115–135 nm) from Ar plasma jet and the effect of nitrogen addition. The intensity of VUV radiation over the radius of the plasma jet was measured. They found that the addition of 5% of N_2_ reduces the integral of VUV emission to approximately 10% of the original value. The radial dependence of VUV emission showed a ring shape with about 1 mm diameter and a minimum in the center. Moreover, Oehrlein et al. [27] investigated the effect of VUV-induced surface modification using an optical window made from pure MgF_2_ that transmits VUV down to the suitable wavelength. They investigated modification of polymethyl methacrylate (PMMA) based 193 nm photoresist (PR193) with 300 nm film thickness and polystyrene (PS) based 248 nm photoresist (PR248) with 400 nm film thickness. Surface modification was investigated by attenuated total reflection Fourier transform infrared spectroscopy (ATR–FTIR) whereas the thickness loss rate measured by ellipsometry. For this investigation different kHz- or MHz-driven argon APPJ plasma sources were used: (1) kHz driven ring-APPJ source, (2) kHz driven pin-APPJ source, (3) MHz driven pin-APPJ source, and (4) kHz driven surface microdischarge source. It was found that the type of the APPJ source is the crucial factor regarding the effect of VUV photons relative to other reactive plasma species to surface modification. Ar fed kHz-driven ring-APPJ source caused the largest VUV surface modification—the highest thickness loss rate when using MgF_2_ filter. If no filter was used, a MHz driven pin-APPJ source caused the highest polymer thickness loss rate. If oxygen was added to Ar feed gas, a reduced VUV effect was observed and explained by the absorption of VUV photons by oxygen molecules. The importance of VUV has also been stressed by Schneider et al. [28]. He has exposed a model a-C: H films to an affluent of helium APPJ with a small admixture of oxygen and found that VUV/UV photons caused hardening of a model a-C: H films on the area in line-of-sight to the jet nozzle, which resulted in slower etching rates of the area directly under the nozzle. 

Onyshchenko et al. [29] performed a two-dimensional mapping of both water contact angle and oxygen concentration on the surface of PET polymer after treatment with the atmospheric pressure plasma jet sustained in high-purity argon. Numerous parameters were varied to estimate the influence of the plasma treatment on the surface composition and wettability: The distance between the dielectric tube and the sample, the exposure time, the discharge power and the gas flow rate. Because of four variable parameters, a limited number of experiments was feasible: Three distances, three exposure times, two powers and two gas flows. Little differences in the surface finish were observed between experiments at different gas flows and discharge powers, whereas, the distance and the treatment time exhibited more pronounced variations of the surface wettability and functionalization. The width of the water contact angle footprints saturated at a rather short treatment time of 20 s at a fixed distance, power and gas flow. The minimal water contact angle of approximately 20° was observed at the shortest distance and the result correlated well with the oxygen concentration as determined by XPS. Detailed investigation on the surface phenomena was not feasible in the work of Onyshchenko et al. because of the large number of independent parameters. In another work, Onyshchenko et al. [30] used a modified design of APPJ with an additional plate at the end of the quartz capillary discharge tube. They found significant improvement in wettability (i.e., much wider hydrophilic footprint area) at short distances between the sample and the nozzle of the capillary discharge tube. At large distances between the sample and the nozzle of the capillary discharge tube, there was only a minor effect on the hydrophilic region created on the PET surface.

The brief survey of the most relevant literature, as shown in Table 1 indicates a variety of techniques, as well as reported results on plasma activation of polymers at ambient pressure. Because of the wide spread of the techniques, it is difficult to draw any correlations. In general, all authors agree that the treatment of polymers with atmospheric pressure plasmas causes significant surface modification, but little explanation for the observed effects is provided. Understanding the reaction mechanisms requires basic knowledge on reactive gaseous species and detailed two-dimensional mapping of the surface wettability at various treatment conditions. A brief study on the influence of the distance between the dielectric tube and the sample, the exposure time, the discharge power and the gas flow rate was already disclosed by Onyshchenko et al. [29,30]. Because there are numerous variable parameters, it is not feasible to vary all of them like the type of gas, the concentration of any additional gases, including impurities, the discharge power, intensity of electrical field, the gas flow and velocity gradients, the distance between a sample and the jet tip, influence of any conductivity of the substrate etc. In this paper, we addressed the wettability at various treatment times and distances between the plasma jet and the samples at practically steady other parameters. The broad ranges of both treatment times and distances enabled a detailed investigation of the evolution of the surface finish. We used a common argon APPJ which contains water vapor as an impurity gas.

## 2. Materials and Methods 

### 2.1. Plasma Treatment

**Figure 1:**
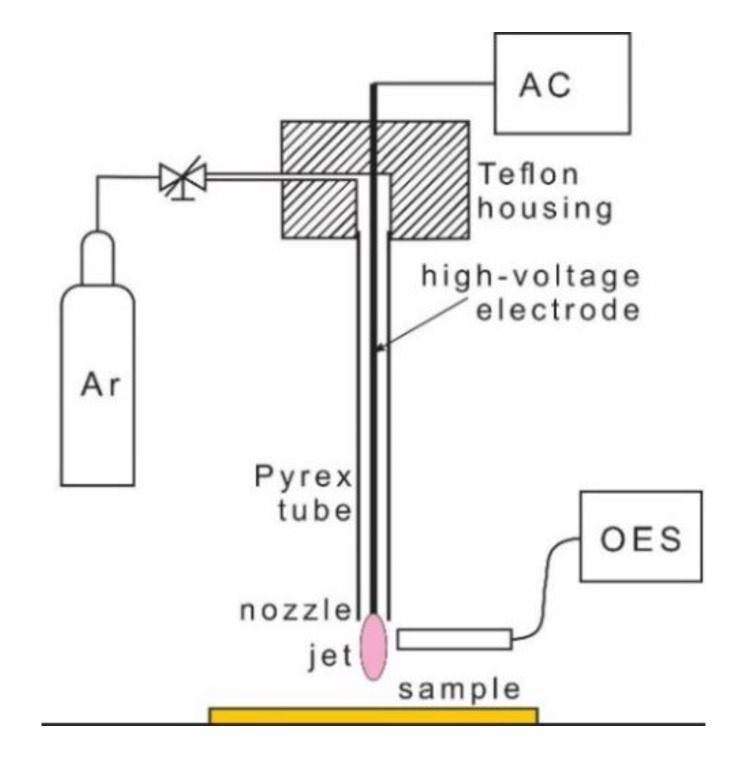
Schematic drawing of the APPJ system used for polymer surface modification.

**Figure 2:**
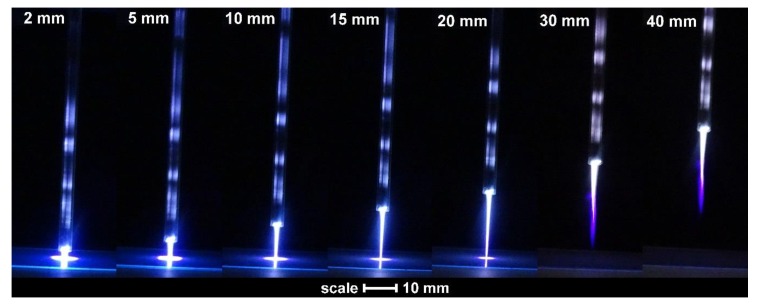
Photos of the plasma jet at various distances of the nozzle from the sample.

Biaxially oriented PET foil (125 µm in thickness) from Goodfellow was cut to squares with a size of 5 × 5 cm^2^ and placed to a wooden substrate holder. Samples were treated in the center of the square with an atmospheric-pressure plasma jet operated in Ar gas at the flow of 1 slm. Schematic illustration of the experimental setup is shown in Figure 1. Plasma was generated along a Pyrex tube with an outer diameter of 4 mm, but the most luminous discharge was observed at the nozzle. A high-voltage electrode (a copper wire) was placed inside the Pyrex tube, as shown in Figure 1. A diameter of the electrode was 0.3 mm, and its length was the same as the length of the Pyrex tube, i.e., 15 cm. A peak-to-peak voltage of 7 kV was applied to the electrode. Plasma was generated with an excitation frequency of 25 kHz using an almost sinusoidal power supply. A visible part of plasma jet extended less than 3 cm from the nozzle of the Pyrex tube (Figure 2). 

As-received polymer samples were placed at different distances to the plasma jet. A distance between the nozzle and the polymer sample was varied between 2 and 40 mm. Individual samples were treated for various treatment times between 0.5 s and 10 min. 

Plasma is a source of various reactive species. To see the role of UV/VUV radiation, an additional experiment was performed where one of the samples was covered with MgF_2_ optical window to transmit only UV/VUV radiation and eliminate other reactive plasma species.

### 2.2. Optical Emission Spectroscopy 

AvaSpec-3648 Fiber Optic Spectrometer (Avantes, Apeldoorn, The Netherlands) was used for characterisation of our APPJ. The spectrometer resolution was 0.5 nm in the range of wavelengths between 200 to 1100 nm. The spectra acquisition time was set to 100 ms. Optical spectra were measured during the treatment of PET foil at various distances of the sample from the nozzle. A collimating lens of the OES spectrometer was placed 2 mm below the nozzle.

### 2.3. Polymer Temperature Measurements 

The average temperature of the polymer surface during plasma treatment was measured with the infra-red IR camera Optris PI 160 (Optris GmbH, Berlin, Germany) working at a wavelength range from 7.5 to 13 µm. The temperature was averaged on the area approximately 7 × 7 mm^2^ around the center of the impact point of the plasma jet with the polymer surface. The surface temperature was measured for two distances of the sample from the discharge nozzle, i.e., 5 and 30 mm. The albedo was fixed at 0.95.

### 2.4. Wettability Measurements 

**Figure 3:**
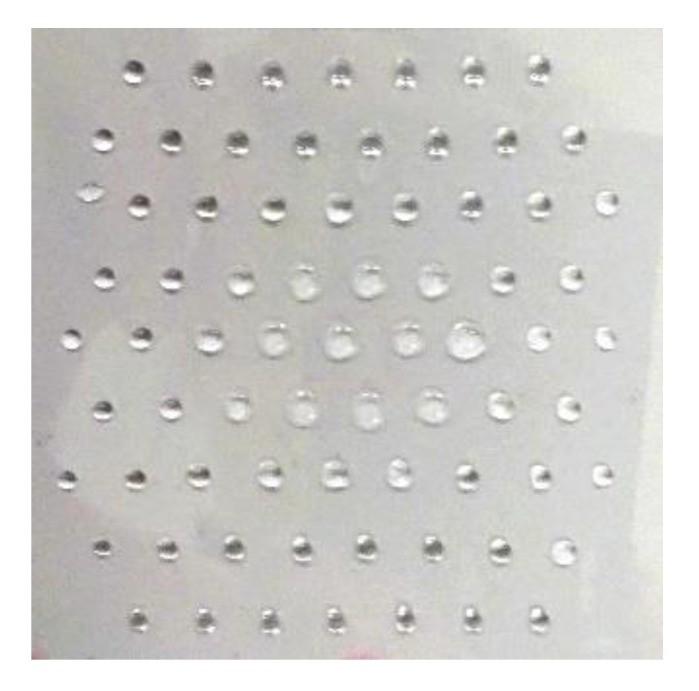
A photo of a grid of water droplets deposited on the polymer surface. In the center, where surface wettability was higher, we can observe wider droplets. The size of the image is 5 × 5 cm^2^.

Mapping of the surface wettability on polymer samples was performed with Drop Shape Analyser DSA-100 (Krüss GmbH, Hannover, Germany). A static contact angle was measured using a sessile drop method. An array of distilled water drops with a volume of 1 µL was applied to the surface with a distance of 5 mm between individual drops. The whole polymer surface was mapped what enabled 2D images of the surface wettability. Each sample of a size of 5 × 5 cm^2^ was, therefore, probed with 73 droplets placed at different spots. A photo of such deposited drops on the sample is shown in Figure 3. The device for measuring the wettability had a stage with fully automatically controlled movement in X and Y direction. By setting the X, Y grid coordinates of the spots on the surface that needed to be measured, it was possible to simultaneously deposit a drop, record an image of this drop and immediately determine the contact angle. Later, all images were manually checked for correct contact angle determination. A time elapsed between the first and the last measured drop was less than 13 min. An ellipse-tangent fitting method was used to obtain contact angles from the shape of the drop.

### 2.5. X-ray Photoelectron Spectroscopy 

XPS characterization of the polymer surface was performed to determine changes in the chemical composition after APPJ treatment using an XPS (TFA XPS Physical Electronics, Münich, Germany). The samples were excited with monochromatic Al Kα_1,2_ radiation at 1486.6 eV over an area with a diameter of 400 µm. Photoelectrons were detected with a hemispherical analyser positioned at an angle of 45° with respect to the normal of the sample surface. To determine the variation of the oxygen concentration over the sample surface, carbon C1s and oxygen O1s spectra were measured in the middle of the treated sample, as well as at various positions over the sample surface (an array of measured points with a distance of 5 mm). In such a way a similar 2D mapping was performed as for wettability measurements. The spectra were measured at a pass-energy of 23.5 eV using an energy step of 0.1 eV. An additional electron gun was used for surface neutralization during the XPS measurements. The measured spectra were analyzed using MultiPak v8.1c software (Ulvac-Phi Inc., Kanagawa, Japan, 2006) from Physical Electronics, which was supplied with the spectrometer. Because of time-consuming experiments, the XPS spectra were acquired only on the limited number of the samples.

### 2.6. Atomic Force Microscopy

The surface morphology was examined by Atomic Force Microscopy (AFM). An AFM (Solver PRO, NT-MDT, Moscow, Russia) was used to determine variations of the surface morphology and roughness around the center of the impact point of the plasma jet with the polymer surface. The measurements were performed in the semi-contact mode. Images with a size of 2 × 2 µm^2^ were recorded.

## 3. Results and Discussion

**Figure 4:**
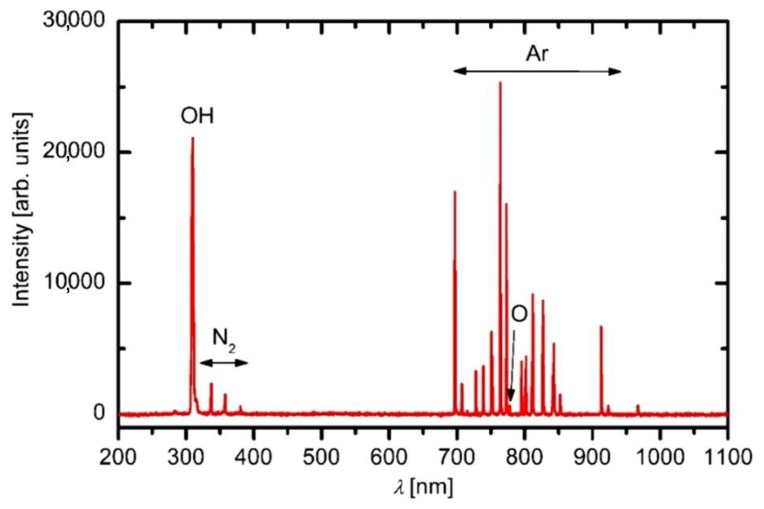
An optical spectrum acquired at a distance between the nozzle and the sample of 2 mm.

**Figure 5:**
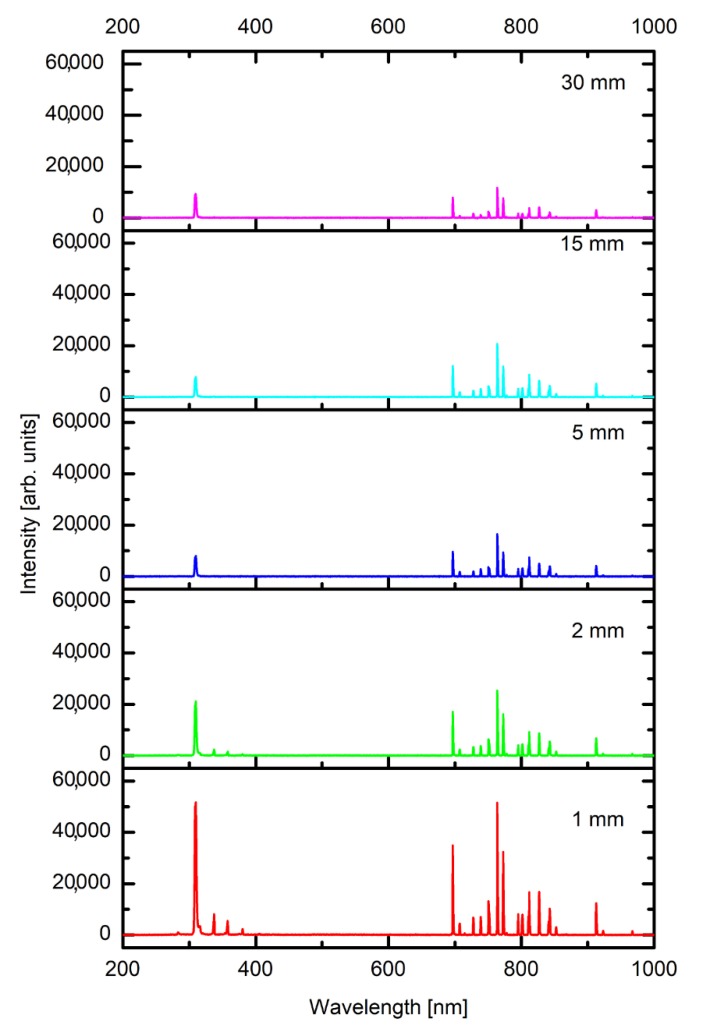
Optical spectra acquired at various distances between the nozzle and the sample. All spectra were acquired with the optical fibre tip placed 2 mm below the nozzle and lenses of an acceptance angle of approximately 3°.

Individual samples were mounted, as shown in Figure 1, and treated by APPJ. Before performing detailed 2D mapping of the surface wettability, the optical spectra of gaseous plasma 2 mm from the nozzle were acquired. A typical OES spectrum is shown in Figure 4. As expected, the OES spectrum is dominated by Ar lines which correspond to transitions of Ar atoms from highly excited states to metastable states. Apart from Ar ions one can observe a small oxygen line at 777 nm and nitrogen molecular band in the near UV range of spectra. The appearance of these spectral features is attributed to mixing of surrounding air with highly excited species of the plasma jet, particularly Ar metastables. A very intensive band is also observed at the bandhead of 309 nm. This band corresponds to the transition of OH radicals from excited to the ground state. The origin is obviously water vapor, which is presented in the ambient air, as well as inside the discharge tube. The intensity of radiation at the same spot, i.e., 2 mm from the nozzle, depends on the distance between the nozzle and the polymer substrate. Figure 5 represents behavior of spectral features at various distances of the sample from the nozzle. The integral intensity is the largest at the smallest distance which is explained by the fact that the light reflected from the sample and the plasma spread across the surface is also captured by the acceptance angle of the collimating lens. This can be seen in Figure 2. 

**Figure 6:**
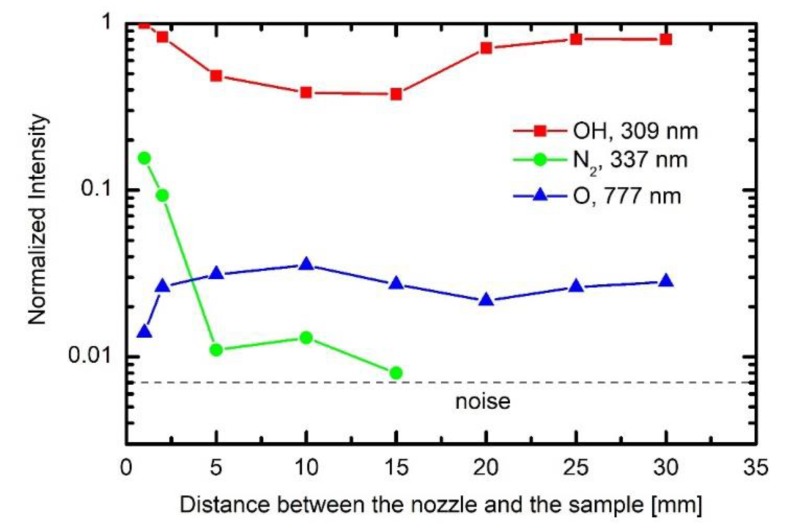
The behavior of normalized spectral features versus the distance between the nozzle and the sample.

More interesting is the behavior of the relative intensities of particular spectral features. Figure 6 represents the intensity of selected spectral features versus the distance between the nozzle and the sample, normalized to the main Ar line at 763 nm. One can observe large differences between nitrogen and OH lines. While nitrogen lines practically vanish over the moderate distance, the OH line still persists even at the largest distance. In fact, the relative intensity of the OH band remains comparable for all distances. The differences in the behavior of the nitrogen and OH lines can be explained by the fact that water retains on any walls of the discharge system throughout the measurement; therefore, the origin of the OH radicals is in the source gas, and not only in the effusion area. Opposite to OH, nitrogen is quickly removed from the discharge tube because it does not condense on the surfaces.

**Figure 7:**
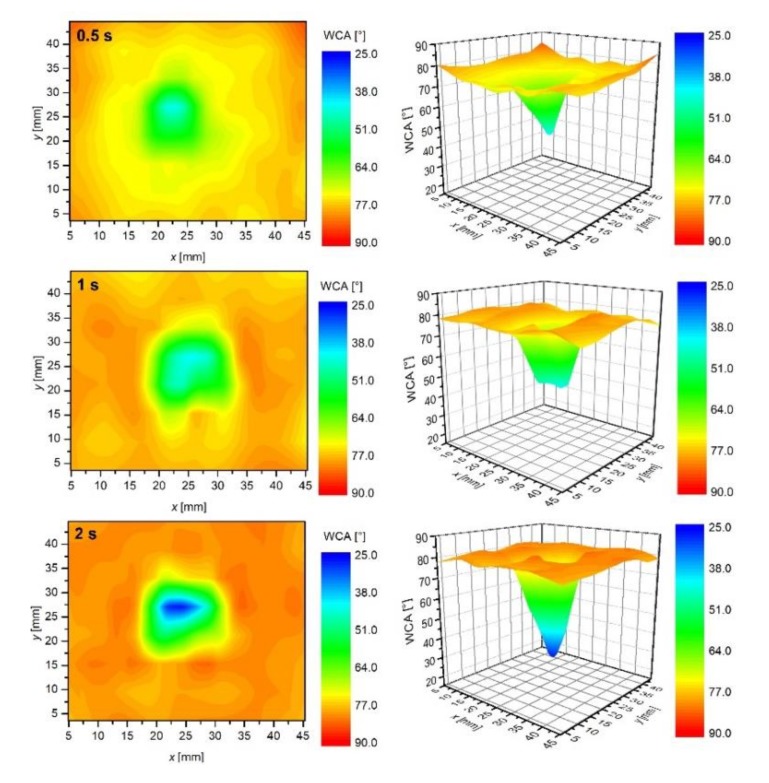

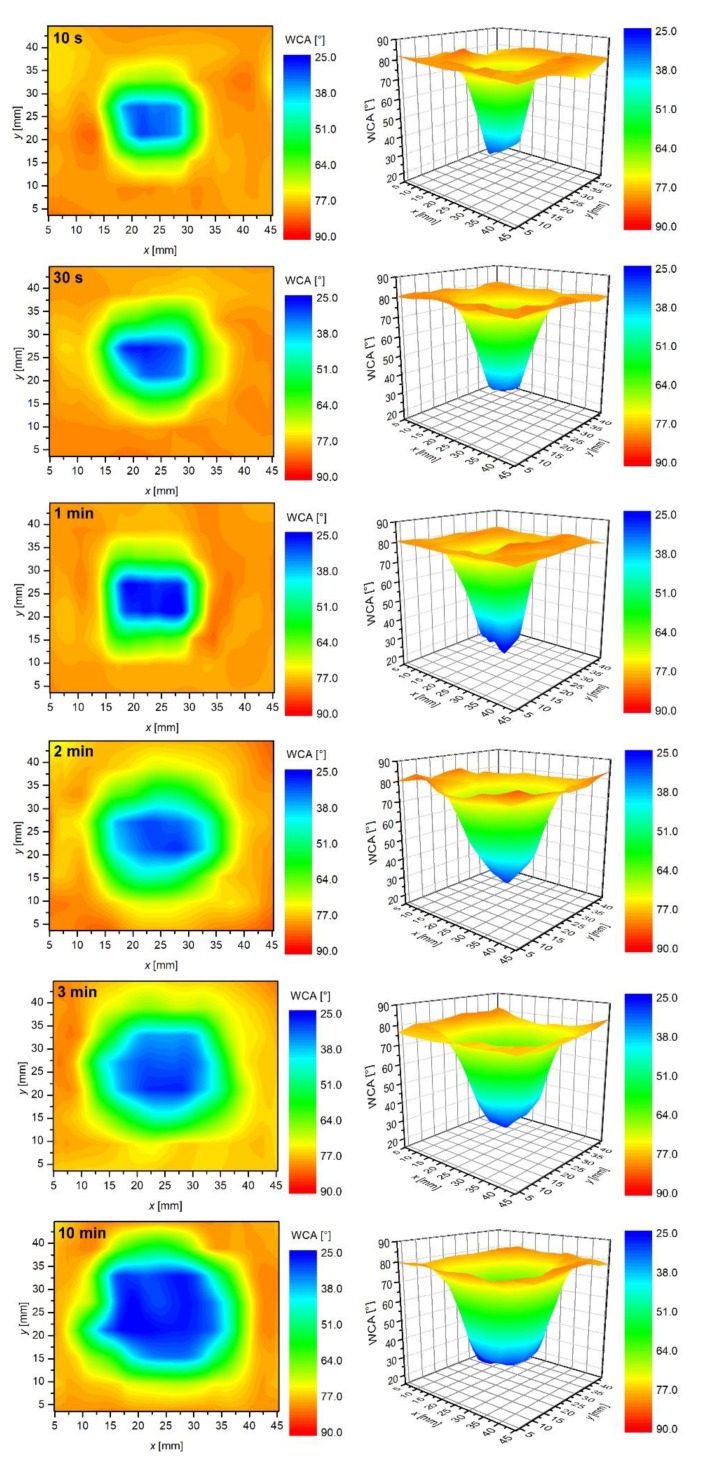
The time evolution of water contact angles on samples placed 5 mm below the APPJ nozzle.

Figure 7 represents results of systematic measurements of the water contact angles on the surface of samples mounted 5 mm below the nozzle. As explained above, the luminous plasma jet was in physical contact with the polymer surface in this case (Figure 2). Plasma jet was focused on the center of the samples. The samples were treated for various periods, and then the surface wettability was measured within less than 15 min after the treatment. 

The uppermost image of Figure 7 corresponds to the plasma treatment time for 0.5 s. We can observe the highest wettability at the axis of the plasma jet. The water contact angle for the sample treated for 0.5 s reaches the minimum of approximately 47° in a small spot in the center of the sample. Away from the spot, the water contact angle increases rather monotonically. The distribution of the water contact angles is best viewed in 3D graphs which are presented in Figure 7. The extreme radial distribution of the surface wettability indicates that the most extensive reactions leading to the improved wettability occur in the center of the plasma jet. The center of a plasma jet in our case is free from nitrogen or oxygen that could diffuse from surrounding atmosphere into the gas jet. The rapid activation of the sample at the center, therefore, cannot be explained by nitrogen or oxygen radicals that appear in the effusion zone of the atmospheric pressure plasma jet. The activation of the sample after 0.5 s treatment should, therefore, be explained by other factors. Among them, and consistent with the literature survey presented in Introduction to this paper, there were energetic Ar particles, OH radicals and UV/VUV radiation. Atmospheric pressure plasma jets sustained at rather low frequencies, and high impedances do not form continuous plasma, but rather appear as numerous streamers. The streamers propagate from the powered electrode in the form of electron avalanches. The driving force of such avalanches is the ionization wavefront. The wavefront propagates in the direction of the highest electrical field, i.e., along the axis of the gas jet. As a result, there is a strong radial gradient of both free electrons and Ar metastables. The flux of Ar ions and metastables on the polymer surface in the case when the polymer is in direct contact with the plasma jet is, therefore, the largest at the axis and decreases rapidly with increased radial distance from the jet axis. The shape of the 3D plot after treatment for 0.5 s (the uppermost image in Figure 7) is in agreement with the radial distribution of the fluxes. From this point of view, we can attribute the initial stage of polymer activation to the effects of energetic Ar particles, in particular, metastables and ions. Both particles transfer their potential energies to the solid material upon impinging. 

While the upper explanation is highly visible from Figure 7, one should also consider the results presented in Figure 4, Figure 5 and Figure 6, in particular, the behavior of the normalized OH “line” in Figure 6. As explained above, such a behavior (almost independent of the distance between the nozzle and the sample) indicates that the water vapor is already presented in the source gas. The origin is desorption of water condensed on any surfaces inside the discharge tube. The molecules dissociate upon interaction with energetic Ar metastables. The OH radicals are clearly visible in the spectra (Figure 4, Figure 5 and Figure 6), whereas, the H atoms are not observed. One explanation for this observation is a rather low electron temperature, which does not allow for significant excitation of hydrogen atoms to radiative states. Another one is the association of H atoms to H_2_ molecules, which are rather poor emitters in the visible range, therefore, they cannot be distinguished from the background of the measured spectra either. The OH radicals are renowned for their oxidation potential. The shape of the 3D graph of the surface wettability after plasma treatment for 0.5 s could, therefore, be explained by chemical activation and formation of oxygen functional groups, due to the existence of OH radicals in the plasma jet. 

**Figure 8:**
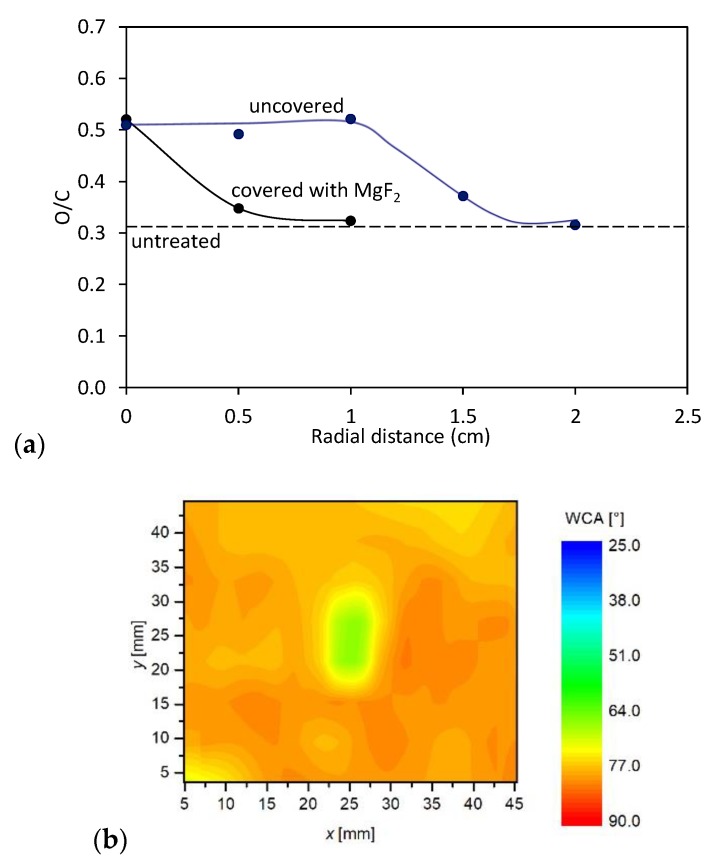
(**a**) Comparison of XPS ratio O/C for the sample covered with MgF_2_ optical window, and thus, exposed to radiation only with the uncovered sample exposed to all reactive species. The sample was placed 5 mm from the nozzle. Treatment time was 1 min. (**b**) Mapping of the surface wettability of the polymer surface covered with a MgF_2_ window. The surface wettability of the corresponding uncovered sample is shown in Figure 7 (1 min).

The third mechanism of surface activation is a bond scission upon the interaction between UV and/or VUV radiation and the surface polymer film. According to Oehrlein et al. [27] and Schneider et al. [28], especially VUV radiation is particularly relevant. VUV radiation in the Ar plasma jet is assigned to Ar_2_* excimer continuum [31,32]. As reported by Oehrlein et al. [27] and explained in Section 1, the type of the APPJ source is a significant factor in the importance of VUV radiation relative to other plasma species in its contribution to surface modification. They found that the relative importance of VUV effects for a kHz ring-APPJ source was over 20%, whereas, for a kHz pin-APPJ and a MHz pin-APPJ it was only about 6% and 1%, respectively. The distribution of any radiation arising from a source is spatially uniform: The photons are emitted in all directions uniformly. However, as mentioned by various authors, VUV effects are reduced in the presence of molecular oxygen [27,28,33], as well as nitrogen [26]. As reported by Schneider et al. [28], the flux of VUV is the most intense just underneath the jet and is absorbed by air at any radial distance from the jet larger than few millimeters. This can also be observed in Figure 8a where the O/C ratio measured by XPS at various radial distances from the axis of the plasma jet is shown. In this case, we have covered one sample with MgF_2_ optical window that transmits only VUV radiation [34], and exposed it to APPJ, at a distance of 5 mm. For comparison, also the XPS composition of the uncovered sample is shown. As observed in Figure 8a, the sample covered with MgF_2_ window was modified only in the center (at the axis of the plasma jet). Because the MgF_2_ window was in contact with the sample surface, the increased oxygen concentration can be explained either by reaction of oxygen adsorbed on the surface with the polymer or by post-treatment reactions of dangling bonds with the ambient atmosphere. With increasing radial distance from the center, a sharp drop in the O/C concentration appeared. This is not observed for the uncovered sample, where a diameter of the modified surface is much larger and comparable with that shown in Figure 8a. In Figure 8b is also shown mapping of the surface wettability of the sample covered with the MgF_2_ optical window (the wettability of the corresponding uncovered sample is shown in Figure 7 (1 min)). The results of surface wettability are in agreement with XPS results–only the small spot on the axis was activated. The minimum contact angle is higher compared to the uncovered sample. This is expected—because the wettability is also influenced by the surface roughness. The uncovered sample was, of corse, exposed to etching effects that were absent in the case of the sample covered with MgF_2_ optical window.

Therefore, at experimental conditions in this work, the VUV radiation is regarded to not play a dominant role in the activation of the polymers, because it cannot explain the formation of such large spots as observed in Figure 7. Therefore, we can conclude that, in our case, the activation by functionalization with OH radicals and/or interaction with long-living energetic Ar species prevails the activation induced by VUV. 

The polymer samples were treated at a distance of 5 mm also for longer times. Figure 7 reveals the temporal evolution of the surface wettability. As expected, the area of a low water contact angle expands with increasing treatment time. Less expected, however, is the fact that the surface activation is limited to a rather small spot even for prolonged treatment times. For example, the sample treated for 10 min at the distance of 5 mm exhibits a surprisingly sharp distribution of the surface wettability. For this sample, the central area of a diameter of about 2 cm is saturated, whereas, the edges of the sample are not affected by plasma treatment at all. In between, there is a rather steep change of the surface wettability, where the water contact angle increases from approximately 25° to over 70° at a radial distance of approximately 0.5 cm. Such a sharp transition is a consequence of several effects, which will be briefly presented below.

As already discussed, the reactive species presented at the axis of the plasma jet cause rapid activation of the polymer sample. The concentration of free electrons, as well as other species formed in the streamers (in particular, within the ionization wavefront) away from the axis, decreases rapidly because of the gas phase collisions. The highest concentration of reactive species is, therefore, concentrated at the axis and decreases with increasing radial distance from the axis. There should be huge radial gradients, so the air molecules effusing the Ar jet, but not reaching the axis cannot be excited to any state that causes polymer activation. If the concentration of reactive species, such as O, NO and N, let alone molecular metastables several cm from the axis were detectable, the polymer sample exposed to plasma for a long time would have been activated entirely. The graphs presented in Figure 7, however, show that the plasma activation is radially limited even for treatment times as long as 10 min. 

Another important doubtless conclusion that can be drawn from results presented in Figure 7 is that the contact angle saturates in the center of the spot even at the treatment time as low as a few seconds. The results show that a rather large spot of a low contact angle appears in the center of the sample already after treating the polymer for 10 s. After saturation is reached, some expansion of the spot with a minimal contact angle is observed with increasing treatment time, but the contact angle does not become lower than about 25° even after minutes of plasma treatment. Such a saturation is typical for many polymers and has been reported by numerous authors using both high and low-pressure discharges, as well as plasma afterglows [35,36]. The saturation is usually explained by a balance between functionalization, etching, as well as possible thermal degradation. Dowling et al. reported that some thermal decomposition might occur at polymers having low glass transition temperature [21].

**Figure 9:**
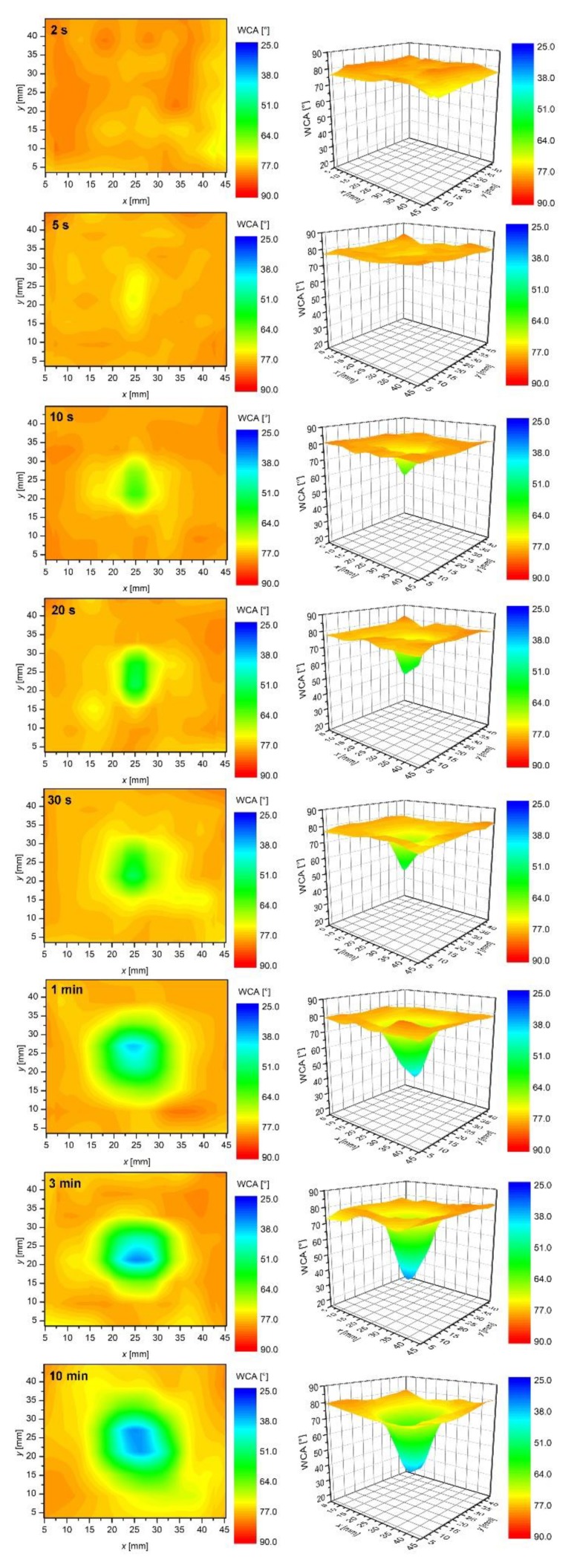
The time evolution of water contact angles on samples placed 30 mm below the APPJ nozzle.

Treatment of this polymer by the plasma jet, therefore, allows for a rapid increase of the surface wettability which is limited to a spot of typical diameter of few cm. Such a surface finish is achievable providing the plasma jet is in direct contact with the polymer sample. In many cases, however, it is not feasible to provide direct contact, for example, in the case of a polymer product of complex geometry. In such cases, the surface wettability may or may not be altered, depending on particularities of the experimental conditions. If a polymer surface is far away from the end of the plasma jet, the concentration of any reactive species is too low to allow for any effect. Particularly interesting are surface alternations in the case where the polymer sample is placed close to the visible end of the glowing plasma jet. Such experiments were also performed, and the results are shown in Figure 9.

The graphs presented in Figure 9, where acquired exactly in the same manner as those in Figure 7 except the distance between the sample and the nozzle was much larger (30 mm); therefore, the visible part of the plasma jet did not touch the samples (Figure 2). The shortest treatment time, shown in Figure 9, is 2 s. No statistically significant modification in the surface wettability is observed for this sample, meaning that a dose of reactive species was too low. For the sample treated for 5 s, however, we can already observe a statistically significant pattern. Right in the center of the sample, there is a spot of a diameter of ~1 mm, where the water contact angle is ~68°. This spot is surrounded by a larger area of a contact angle of ~78°. The observed feature indicates the initial stage of the sample activation. A double time (10 s) already reveals a rather large spot of the activated material of the contact angle ~52°. The spot diameter increases with increasing treatment time, but the contact angle in the center of the spot does not drop below 39° even after 1 min of plasma treatment. Contact angles lower than 39° are detectable only after prolonged treatment. The results presented in Figure 9, therefore, reveal that the activation not only takes longer time than in the case of direct exposure to the glowing plasma, but the minimal contact angle of 25° appears only after prolonged treatment time (10 min). Because the visible plasma jet expands only up to approximately 25 mm from the nozzle, i.e., 5 mm from the sample surface (Figure 2), the concentration of reactive species that owe their existence to electron impact events is negligible as compared to the glowing jet. The reactive species found in the early afterglow are definitely Ar metastables, and there could also be some long living chemically reactive species, such as nitric oxides and ozone [10,37]. There is a strong gradient in the concentration of OH radicals at the edge of the plasma jet [38]. 

The sample treated at a distance of 30 mm from the nozzle for 10 min exhibits a similar wettability as a sample treated directly with the plasma jet for 10 s. The ability for activation of the PET surface just outside the plasma jet is, therefore, about two orders of magnitude smaller than in the glowing plasma. The practical consequence of the observed effects is that any treatment of products of complex geometry using such a simple device as our APPJ is impractical because the required treatment time to saturate the surface wettability is too long even for a short distance between the end of the glowing plasma jet and the substrate.

**Figure 10:**
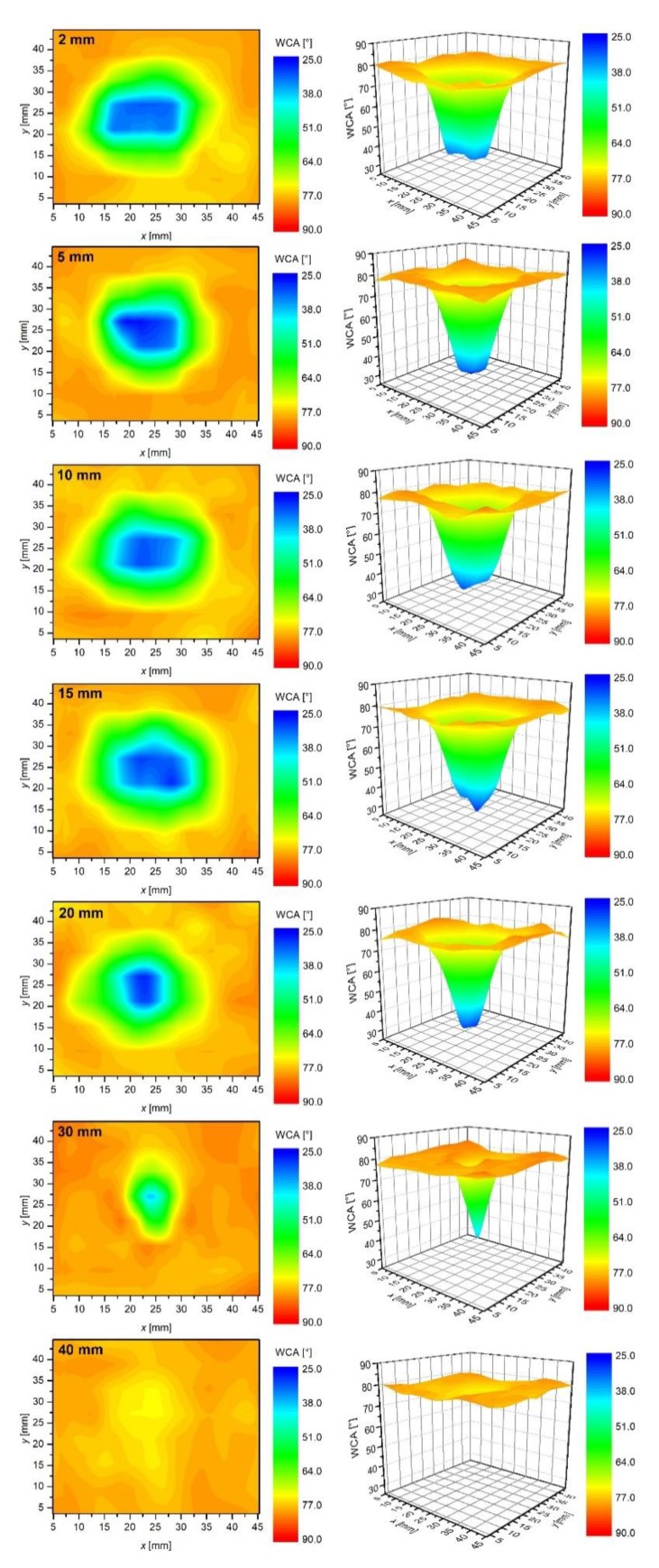
The water contact angles on samples treated for 30 s at different distances between the APPJ nozzle and samples. The visual length of plasma jet is below 30 mm from the nozzle.

The upper discussion is proved by additional results presented in Figure 10. In that set of measurements, the plasma-on-time was fixed to 30 s, but the distance between the nozzle and the sample was varied between 2 and 40 mm. Figure 10 reveals little difference between the samples treated at various distances up to 20 mm, i.e., when the plasma jet was in direct contact with a sample surface. For the case of 30 mm, there was a reasonable activation, but at 40 mm we observe no statistically significant modifications of the polymer surface. The huge difference between the last two images, shown in Figure 10, indicates that the concentration of reactive species capable of activation of the polymer surface about 1 cm away from the glowing plasma jet is negligible. 

**Figure 11:**
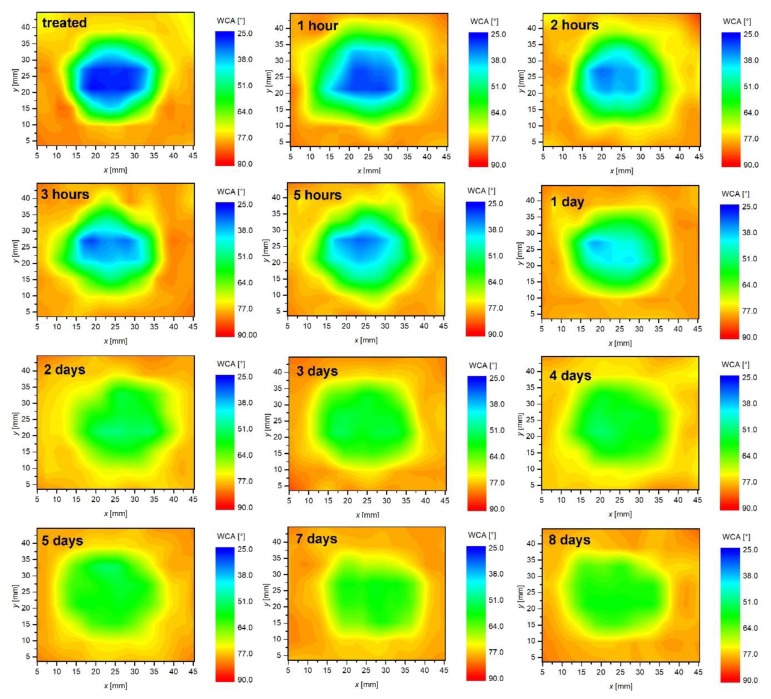
2D evolution of water contact angles versus aging time.

**Figure 12:**
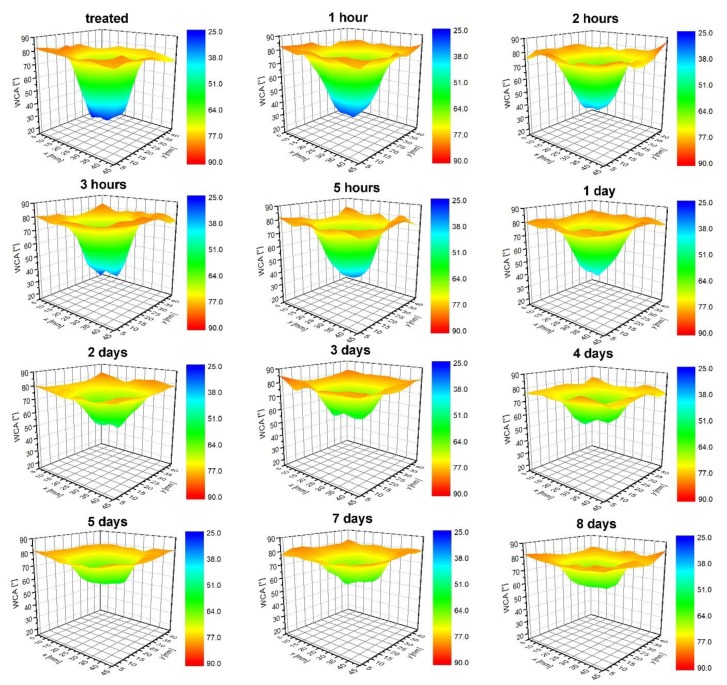
3D evolution of water contact angles versus aging time.

**Figure 13:**
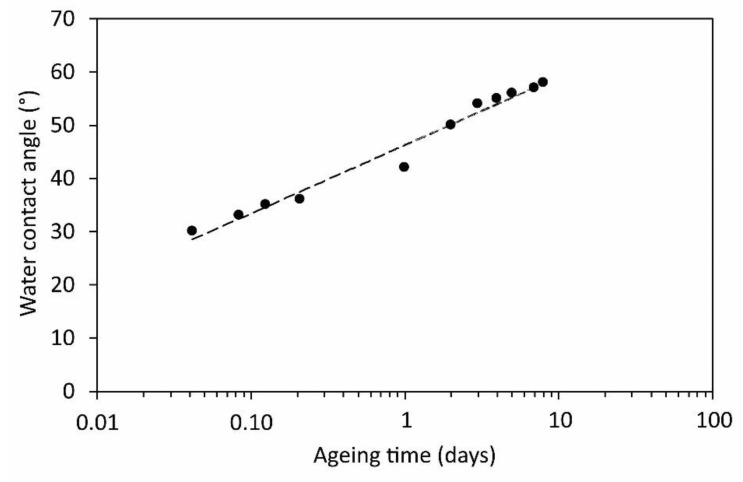
The evolution of the minimum water contact angles versus aging time.

Polymers like PET are moderately hydrophobic; therefore, any hydrophilicity is thermodynamically unstable. The hydrophilicity decreases spontaneously upon aging. The effect is known as “hydrophobic recovery”. The aging of a sample treated at a distance of 5 mm, and for 3 min was, thus, measured and is shown in Figure 11 and Figure 12. Figure 11 and Figure 12 represents the evolution of water contact angles upon aging at room temperature for different periods. The area of a low contact angle in the middle of the modified zone ages preferentially. It is clearly visible that the zone of originally low contact angles ages rather quickly, whereas, the edges remain almost perfectly intact. After several days, the water contact angle assumes the same value over the entire area of the modified zone. The area of a higher wettability, therefore, ages faster than the area of a moderate wettability. This observation is in agreement with several reports on the hydrophobic recovery of various polymers: The aging is faster for highly activated polymers. The aging roughly follows the logarithmic dependence as revealed from Figure 13, which shows the water contact angle in the center of the modified zone versus the aging time. 

**Figure 14:**
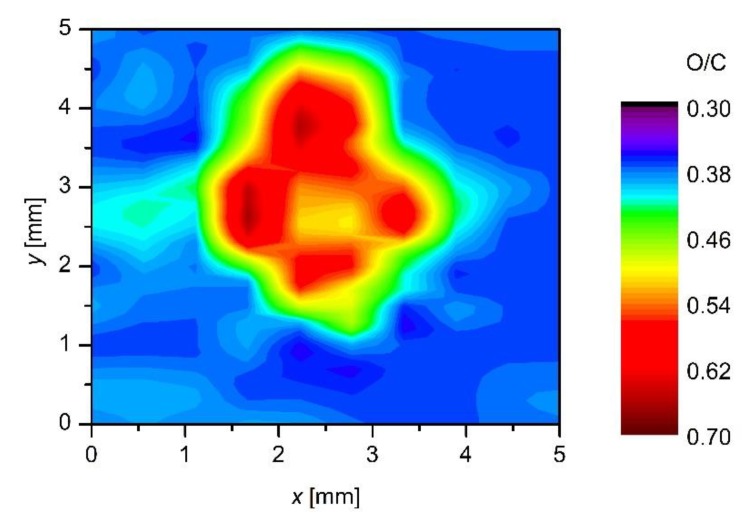
2D XPS mapping of the O/C ratio of a sample treated for 3 min at a distance of 5 mm from the nozzle.

Although the wettability is well known to depend on the surface morphology and concentration of functional groups, we performed 2D XPS mapping to prove the origin of the surface activation. Because the XPS mapping is time consuming, we only performed the measurements for the sample treated 5 mm from the nozzle for 3 min. The corresponding wettability for this particular sample is shown in Figure 7 (marked with 3 min). The ratio between oxygen and carbon as calculated from XPS survey spectra of this sample is shown in Figure 14. Although the spot size as detected by XPS is somehow smaller (because of the limits of the XPS device, a grid of measured points was not as dense as in the case of WCA measurements), the major features are analogous. In both cases, there is a rather large spot of a diameter just below 2 cm, where the surface is saturated; i.e., in Figure 7, the saturation is observed for the case of the water contact angle, whereas, in Figure 14 in terms as O/C ratio. The surface activation as elaborated in Figure 7, Figure 9 and Figure 10, therefore, arise from functionalization of the polymer surface with oxygen rich functional groups. In both cases (surface wettability and O/C ratio), a rather sharp interphase between saturated area and the not affected area is observed. As already discussed above, such a sharp interphase is a consequence of very strong gradients of reactive species at the edge of the plasma jet.

**Figure 15:**
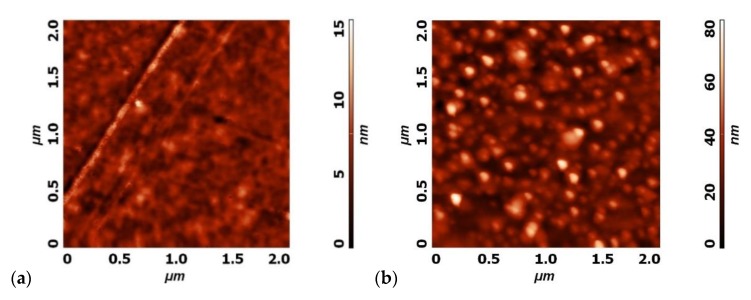
AFM images of: (**a**) The untreated PET foil and (**b**) the PET foil treated for 10 min at a distance of 5 mm.

As already reported, the surface morphology of the sample may change as a result of plasma treatment [16]. Figure 15 shows typical AFM images obtained over a surface area of 2 × 2 µm^2^. The left image is for the untreated sample, and the right one for the sample treated for 10 min at a distance of 5 mm from the nozzle. The plasma-treated sample assumes a morphology resembling nanofeatures. 

**Figure 16:**
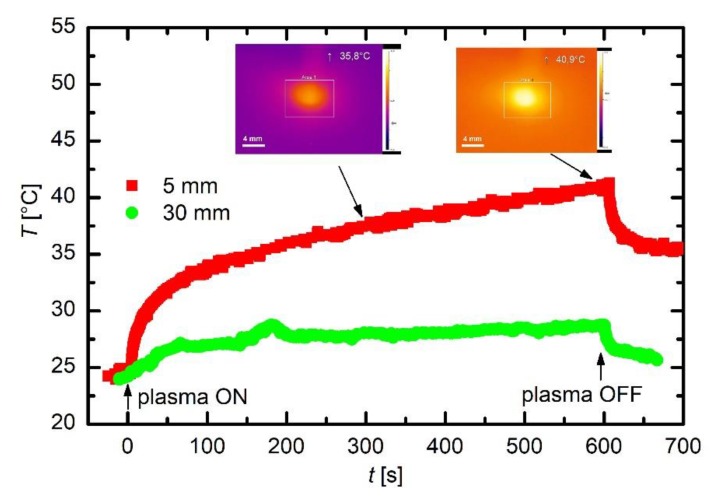
Evolution of sample temperatures as determined with the IR Pyrometer for the case when samples were placed 5 and 30 mm from the nozzle of the discharge tube.

Finally, it is worth mentioning that there are always exothermic reactions on the surface of a material treated by gaseous plasma despite the fact that plasma jets as adopted in this study are cold. As discussed above, the list of exothermic reactions includes oxidation of the surface because of interaction with OH and some other radicals, as well as absorption of light quanta, neutralization of charged particles and relaxation of metastables. To estimate the heat load, we measured the surface temperature versus treatment time with the IR pyrometer. Although the albedo may change during the measurement because of variation of surface morphology, composition and structure, we took into account a constant emissivity in the range of wavelengths probed with the pyrometer. Figure 16 shows the temperature evolutions versus treatment time for the cases when the PET sample was positioned 5 and 30 mm from the nozzle of the discharge tube. For a rather large distance (i.e., 30 mm), the surface temperature as probed with the pyrometer increased for few degrees centigrade; therefore, the thermal load is regarded marginal. For a short distance (i.e., 5 mm), however, the surface temperature increased to approximately 35 °C in the first 100 s. Thereafter, it does not stabilize, but keeps increasing almost linearly with treatment time. The linear increase is explained by a huge thermal capacity of the sample holder as compared with the sample itself. The heat is used for increasing the temperature of the sample holder; thus, the sample temperature remains reasonable, i.e., 40 °C for 10 min of treatment. Such a low temperature has not been reported by many authors who have used a similar experimental setup. For example, Dowling et al. [21] reported temperatures exceeding 60 °C. The discrepancy is explained by differences in experimental conditions, in particular, the thermal contact between the substrate and the sample holder, as well as thermal properties of the holder. After turning off the discharge, the sample temperature quickly dropped to approximately 35 °C and then it slowly decreased as revealed from Figure 16. The fact that the sample temperature did not drop to initial temperature (i.e., 24 °C) is explained by a huge thermal capacity of the sample holder providing the albedo has not changed. 

## 4. Conclusions

Results of systematic 2D mapping of the surface wettability enable an insight in processes responsible for surface activation of PET polymer using a simple atmospheric pressure plasma jet. The discharge tube was only flushed with Ar gas to get rid of permanent gases, such as nitrogen, oxygen and CO_2_, but the water remained on any surfaces as a result of the humidity of the laboratory atmosphere. The OES spectra showed significant radiation from the OH radicals. Unlike nitrogen bands, which were observed only when the sample was close to the exhaust of the dielectric discharge tube, OH radiation persisted even for long distances of the sample from the nozzle. This observation indicated that the OH origin was water vapor which was slowly desorbing from the surfaces of the discharge tube and/or electrode when the sample was treated with Ar plasma. The wettability of samples exhibited saturation at the water contact angle of approximately 25° as long as the samples were exposed to the glowing plasma at a short distance from the nozzle. At longer distances of the sample from the nozzle, the minimal contact angle of 25° was achieved at a much longer time which is impractical. A rather sharp interphase between the highly wettable area saturated with oxygen functional groups and the surrounding unaffected area was observed in all cases. The results were explained with both radial and axial gradients of reactive species useful for activation of the polymer materials. Almost perfect overlapping of the area saturated with oxygen functional groups with highly wettable area suggests that the major reactants causing activation of the polymer at these experimental conditions are OH radicals.

## Figures and Tables

**Table 1:** Literature overview. APPJ, atmospheric-pressure plasma jets

Reference	APPJ Parameters	Gas	Sample Distance	Variables	Methods for Plasma Characterisation	Methods for Sample Characterisation	Polymer	Wettability before and after Treatment
[16]	5 kH, 20 W, 12 kV DC, treatment time: 1, 5, 10 min	He, Ar or mixture with 1% O_2_ or N_2_	10 mm	Gas type	/	XPS mapping SEM	PE	
[17]	AC 37 kHz, 4.4 W, gas flow 1.3 L/min, treatment time: Up to 90	Ar	2–3.5 cm	Treatment time, distance	Current-voltage waveforms	WCA line profile, XPS AFM	PET PP PE	78° → 25° 102° → 52° 94° → 36°
[20]	13.56 MHZ, gas flow 1 slm, treatment time up to 30	He with up to 1% of O_2_ admixture	3–50 mm	Treatment time, distance, oxygen admixture	/	WCA	PP	95° → 50°
[18]	Rotating plasma jet (Openair system, Plasmatreat GmbH), torch speed: 10–30 m/min, DC arc plasma, 270–300 V, 19–22 kHz, pulsed mode, stage moving speed 5–50 m/min	Compre-ssed air	5–10 mm, optimum at 6.6 mm	Distance, torch speed	OES, gas temperature	WCA, XPS	PDMS	109° → <10°
[21]	Openair system (Plasmatreat GmbH), 25 kHz, flow rate 76,6 L/min, pulse peak height 2–5 kV, stage moving speed 30 m/min, treatment time ~5 ms	Compre-ssed air	16 mm	Pulse plasma cycle time	OES	WCA, aging, AFM, XPS, polymer temperature	PP PS PC	90° → 70° 93° → 20° 83° → 30°
[22]	Openair system (Plasmatreat GmbH), 17–22 kHz, pulse peak height 5 kV, flow rate 17 L/min, stage moving speed 100 m/min, treatment time < 0.1 s	Air or nitrogen	3 mm	Gas type	OES	WCA, XPS	PE	93° → 22° (for air) 93° → 41° (for N_2_)
[19]	Flow 1.25 slm, scanning velocity 0.08-4.5 m/min, treatment time 78 ms	Ar	5–45 mm	Distance	Current-voltage waveforms	WCA, XPS, aging	PE	104° → 28°
[27]	Different APPJ sources: (1) kHz driven ring-APPJ source, (2) kHz driven pin-APPJ source, (3) MHz driven pin-APPJ source, (4) kHz driven surface microdischarge source	Ar with or without O_2_ addition	Up to 3 cm	Different APPJ sources	optical window for VUV, COMSOL modelling	ATR-FTIR, ellipsometry	PMMA based 193 nm photore-sist (PR193) and PS based 248 photore-sist (PR248)	
[29]	AC 60 kHz, peak-to-peak voltage 7 or 10 kV, discharge power 3.1–5.7 W, gas flow: 1 and 3 slm, treatment time 5 to 20 s	Ar	2, 10 and 15 mm	Treatment time, distance, discharge power, gas flow	OES	WCA, XPS	PET	87° → 22°
